# Genomic Epidemiology of Healthcare-Associated Respiratory Virus Infections

**DOI:** 10.1017/ash.2025.231

**Published:** 2025-09-24

**Authors:** Vatsala Rangachar Srinivasa, Marissa Griffith, Alexander Sundermann, Kathleen Shutt, Kady Waggle, Graham Snyder, Daria Van Tyne, Lora Pless, Lee Harrison

**Affiliations:** 1 University of Pittsburgh; 2 UPMC; 3 UPMC/University of Pittsburgh

## Abstract

**Background:** Investigation of transmission of respiratory viruses (RV) in healthcare setting is understudied. To investigate the transmission dynamics of common healthcare-associated RV infections, we performed retrospective whole genome sequencing (WGS) surveillance of rhinovirus, influenza, human metapneumovirus (HMPV), and respiratory syncytial virus (RSV) at one children’s and two adult teaching hospitals in the Pittsburgh area. **Methods:** From Jan 2, 2018 to Jan 4, 2020, nasal swab specimens positive for rhinovirus, influenza, HMPV or RSV were collected from patients who had been hospitalized for ≥3 days. Specimens with qPCR Ct values ≤30, HMPV specimens were sequenced using tiled PCR amplicons regardless of their qPCR Ct value. Genomes passed WGS QC if ≥90% of the genome had ≥10× average coverage depth. High-quality genomes were assessed for genetic relatedness using ≤3 single nucleotide polymorphisms (SNPs) as a cut-off. Review of patient health records was performed on all genetically related clusters to identify common epidemiological connections. **Results:** We collected 436 rhinovirus (n = 291), influenza (n = 50), HMPV (n=47) and RSV (n=48) specimens from 360 patients. Of these, 55% (197/360 patients) were from the children’s hospital and 45% were from the two adult hospitals. Patients ranged in age from 14 days to 93 years old, 61% were male, and 74% were white. We sequenced 61.2% (178/291) of rhinovirus, 78% (39/50) of influenza, and 92% (44/48) of RSV specimens that met qPCR criteria. Among these, 63.5% (113/178) of rhinovirus, 87% (34/39) of influenza, and 89% (39/44) of RSV genomes passed WGS QC. Additionally, 79% (37/47) of the HMPV genomes passed WGS QC. We identified 13 genetically related clusters (n=5 rhinovirus; n=2 influenza; n=3 RSV; n=3 HMPV) containing 34 patients and ranging in size from 2-5 patients per cluster. We identified common epidemiological links between 56% (19/34) of clustered patients. Of these, 63% (12/19) of patients had a same-unit stay, 11% (2/19) shared a common provider, and 26% (5/19) had overlapping hospital stays. On average, genetically related clusters spanned a duration of 17 days (range: 0−55 days). **Conclusion:** WGS offered valuable insights into RV transmission dynamics in hospitals, which until now have not been rigorously studied. While healthcare-associated RV transmission is common, absence of epidemiological links in 44% of genetically-related cases and the distribution of cluster durations, given the incubation period, highlights the complex transmission dynamics.

